# Genetic variation in surfactant protein-A2 alters responses to ozone

**DOI:** 10.1371/journal.pone.0247504

**Published:** 2021-02-22

**Authors:** William P. Pederson, Jaime M. Cyphert-Daly, Robert M. Tighe, Loretta G. Que, Julie G. Ledford

**Affiliations:** 1 Department of Physiology, University of Arizona, Tucson, Arizona, United States of America; 2 Department of Medicine, Duke University, Durham, North Carolina, United States of America; 3 Asthma and Airways Disease Research Center, Tucson, Arizona, United States of America; 4 Cellular and Molecular Medicine, University of Arizona, Tucson, Arizona, United States of America; University of Alabama at Birmingham, UNITED STATES

## Abstract

**Background:**

Increased exposure to Ozone (O_3_) is associated with adverse health effects in individuals afflicted with respiratory diseases. Surfactant protein-A (SP-A), encoded by *SP-A1* and *SP-A2*, is the largest protein component in pulmonary surfactant and is functionally impaired by O_3_-oxidation.

**Objective:**

We used humanized SP-A2 transgenic mice with allelic variation corresponding to a glutamine (Q) to lysine (K) amino acid substitution at position 223 in the lectin domain to determine the impact of this genetic variation in regards to O_3_ exposure.

**Methods:**

Mice were exposed to 2ppm O_3_ or Filtered Air (FA) for 3 hours and 24 hrs post-challenge pulmonary function tests and other parameters associated with inflammation were assessed in the bronchoalveolar lavage (BAL) fluid and lung tissue. Additionally, mouse tracheal epithelial cells were cultured and TEER measurements recorded for each genotype to determine baseline epithelial integrity.

**Results:**

Compared to FA, O_3_ exposure led to significantly increased sensitivity to methacholine challenge in all groups of mice. SP-A2 223Q variant mice were significantly protected from O_3_-induced AHR compared to SP-A^-/-^ and SP-A2 223K mice. Neutrophilia was observed in all genotypes of mice post O_3_-exposure, however, SP-A2 223Q mice had a significantly lower percentage of neutrophils compared to SP-A^-/-^ mice. Albumin levels in BAL were unchanged in O_3_-exposed SP-A2 223Q mice compared to their FA controls, while levels were significantly increased in all other genotypes of O_3_-exposed mice. SP-A 223Q MTECS has significant higher TEER values than all other genotypes, and WT MTECS has significantly higher TEER than the SP-A KO and SP-A 223K MTECS.

**Significance:**

Taken together, our study suggests that expression of a glutamine (Q) as position 223 in SP-A2, as opposed to expression of lysine (K), is more protective in acute exposures to ozone and results in attenuated O_3_-induced AHR, neutrophilia, and vascular permeability.

## Introduction

Air pollution has become a critical problem not only for climate change and global conservation efforts, but also for public health [1]. Worldwide, millions of individuals are exposed to air pollution levels that exceed the recommended quality standards. The average adult breathes about 11,000 liters of air per day, so the lungs are under constant insult from these airborne pollutants, in addition to combating pathogens, allergens, and noxious gases. Although seemingly overwhelming, the lungs implement several immune mechanisms to combat this problem, one of which is the production of pulmonary surfactant [2].

Pulmonary surfactant lines the airways, and contains four known surfactant proteins (SP-A, B, C, and D), among other lipoproteins. SP-B and SP-C are important for maintaining adequate surface tension within the alveolar sacs, keeping them open and allowing diffusion of oxygen into the blood. SP-A and SP-D are known for their immune and host defense properties, aiding in the protection from invading contaminants and limiting an overzealous immune response. SP-A is the most abundant protein in pulmonary surfactant and is generally thought to play a more immune regulatory role than the other surfactant proteins [3].

Human SP-A is made up of products from two distinct genes, *SFTPA1* and *SFTPA2*. Previous studies have shown that structurally under normal conditions, SP-A forms an octadecamer containing six trimeric subunits, each of which is made up of two SP-A1 proteins, and one SP-A2 protein. These proteins, SP-A1 and SP-A2, have differences at only 10 amino acid residues. Previous studies have shown that when SP-A is exposed to ozone structural and functional changes occur [4].

More specifically, SP-A2 has been shown to be more susceptible to the harmful effects of ozone exposure than its SP-A1 counterpart [4]. While O_3_ is a common, reactive gas found in the upper atmosphere, ground level ozone is associated with increased pollution levels. O_3_ is one of the principal components of smog and is formed by the reaction with sunlight via a photochemical reaction of pollutants that are available in the air from vehicle emissions and industrial contamination. The highest levels of ozone pollution occur during periods of sunny weather. Elevated ground level O_3_ exposure has been shown to cause negative health effects in individuals with respiratory diseases such as asthma and COPD. As a highly reactive compound, O_3_ reacts with unsaturated carbon-carbon double bonds, amino acids, and other chemical structures. Specifically, O_3_ can react with SP-A and has been demonstrated to alter morphological characteristics as well as functional properties [4]. In addition, a specific genetic variant of SP-A2 (rs1965708), which corresponds to the Q (Gln) to K (Lys) amino acid substitution at position 223 of the lectin domain, has been associated with lower lung function and worse asthma control [5]. In the general population, the prevalence of one allele of this specific genetic variation is approximately 20–25%, while the prevalence of being homozygous is about 8%. Interestingly, the prevalence of one allele in African American populations is nearly 35%, with a homozygous rate of about 12% [6]. With rates in variation this high, this specific genetic variation has the potential to impact a significant proportion of the population.

In this study, we wanted to examine if this genetic variation at position 223 SP-A2, alters the physiological and immunological response to acute ozone exposure. Using genetically engineered mice expressing human SP-A2 with the specific amino acids in question (SP-A2 223Q and SP-A2 223K), we discovered that SP-A2 223Q confers protection to an acute ozone challenge over SP-A2 223K in both inflammatory responses and lung function. Based on baseline TEER measurements of epithelial cells cultured at an air-liquid interface, epithelial integrity of cells expressing SP-A2 223Q may contribute to a more protective phenotype to environmental exposures.

## Materials/Subjects and methods

### Mouse models

All experiments were done in accordance with Duke University and The University of Arizona institutional animal care and use committees on IACUC approved animal protocols. SP-A humanized transgenic mice were generated as previously described [7]. SP-A^-/-^ mice on C57BL/6 background were bred in-house and wild-type (WT) C57BL/6 mice were purchased from Jackson Laboratories (Bar Harbor, ME) and bred in house for experiments. Age-matched (approximately 8–10 weeks) male mice were used for experiments.

WT, SP-A^-/-^, humanized SP-A2 223Q and SP-A2 223K male mice (S1 Table) were exposed to either 2 ppm O_3_ or Filtered Air (FA) for 3 hours. All exposures were conducted between 9:00AM and 12:00PM. Ozone exposure at 2ppm for 3 hours is intended to mirror human ozone exposure and is based on previous data and published literature [8–10]. According to the United States Environmental Protection Agency (EPA), ground-level ozone concentrations are considered high, and potentially harmful to humans, at 0.07 ppm over an 8-hour period [11]. However, studies have shown that the deposition fraction of rodents is significantly lower than that of humans, indicating that a dose 4–5 time greater would be required in rodents to create an equivalent response to humans [12]. Additionally, previous studies have compared responses in mice with 1ppm and 2 ppm ozone exposures. The mice exposed to 1ppm of ozone for 3 hours had minimal inflammatory response, while the mice exposed to 2ppm of ozone had significantly more inflammation [8]. With these data in mind, a higher ozone exposure concentration of 2 ppm over 3 hours ensures a robust respiratory response, adequate for modeling disease.

Twenty-four hours post challenge, mice were analyzed on the Flexivent system (SCIREQ Inc., Montreal, Qc, Canada). Briefly, mice were anesthetized via injection of urethane (1 g/kg) followed by pancuronium bromide (0.8 mg/kg, Sigma P1918) as a paralytic. Following a brief equilibration period under default mechanical ventilation settings (150 breaths/min, tidal volume of 10 ml/kg, and a PEEP of 3 cmH_2_O), two maneuvers (inflation to a standard pressure of 30 cmH_2_O over a 3 second and holding for an additional 3 seconds) were performed to open closed lung areas and standardize lung volume history. Single frequency (Snapshot-150; 2.5Hz) and broadband (Quick Prime-3; 1–20.5 Hz) FOT measurements were alternated a few seconds apart for a total of 12 measurements per perturbation over a period of approximately 3 minutes. This protocol was repeated a total of four times with increasing concentrations of aerosolized methacholine (0, 12.5, 50, 100 mg/ml) as previously described [13]. Following lung function assessment, of which airway resistance and elastance were direct readouts from the Flexivent machine, mice were euthanized and the lungs lavaged three times with PBS (0.1 mM EDTA). The right lung lobes of these animals were then excised and stored in cryotubes at -80°C for further analysis. Differential cell counts were analyzed from the lavage fluid after H&E staining with Easy III Stain Kit (Azer Scientific, Morgantown, PA, United States). Cell viability was assessed by Trypan blue exclusion.

### Histological analysis

Mice were euthanized after pulmonary function tests as described above. Left lung lobes were dissected and immersed in 10% buffered formalin for fixation. After 3 days, the lung lobes were transferred from formalin to 70% ethanol, then routinely processed and paraffin embedded for staining. Stained sections photographed at 40X magnification.

### ELISA

Albumin and keratinocyte chemoattractant (KC) were analyzed in the BAL by ELISA according to manufacturer’s protocols. For albumin measurements, samples were diluted 1:500 for detection with the mouse albumin antigen ELISA Kit (Molecular Innovations, Lot:MSAKT-218). For KC measurements, samples were neat for detection with the mouse KC ELISA Kit (RayBiotech Inc.; Cat# ELM-KC—Lot# 060518 0476). Levels were detected on a plate reader (BioTek SYNERGY HTX) at 450 nm wavelengths.

### RT-PCR

Mouse tissues were collected into 1 ml of TRI Reagent^®^ (Sigma). RNA was isolated using the standard TRI reagent/chloroform extraction method. DNA was synthesized from 1 μg of total RNA using Bio-Rad^™^ cDNA Synthesis kit. Reverse transcription polymerase chain reaction (RT-PCR) was performed using Bioline 2x SensiFAST SYBR no-ROX mix. The samples were analyzed for expression levels of mouse KC using forward and reverse primers specific to the gene (forward 5’ TGG CTG GGA TTC ACC TCA AGA AC 3’, reverse 5’ TGT GGC TAT GAC TTC GGT TTG GGT 3’). The relative levels of expression obtained were normalized to the mammalian housekeeper gene Cyclophilin using primers specific to the gene (forward 5’ AGC ACT GGA GAG AAA GGA TTT GG 3’, reverse 5’ TCT TCT TGC TGG TCT TGC CAT T 3’).

### Mouse tracheal epithelial cell harvest and growth conditions

Mice of each genotype (WT, SP-A^-/-^, SP-A2 223Q, and SP-A2 223K) were euthanized with Urethane and tracheas were removed and placed in Ham’s F-12 media on ice. After all the tracheas had been collected, they were moved into a sterile environment within a Biosafety Cabinet. Here, tracheas were cleaned by removing any excess tissue, including connective tissues, vasculature, muscle, and nerves. The tracheas were then dissected longitudinally to expose the epithelial cells on the inner surface, before being placed in Ham’s F-12 Media containing 0.1% Protease solution (Sigma) and incubated at 37°C for 45 minutes. Protease activity was halted with the addition of Fetal Bovine Serum (FBS) (Atlanta Biologics). The tracheas were removed from solution and transferred to a petri dish with Ham’s F-12 media, where the mucosal lining was scraped off. The cells were collected and transferred to a 15mL conical tube, where they separated from solution via centrifugation at 900rpm for 5 minutes, at 4°C. The cell pellet was then resuspended in 5mL of Versene (Gibco/Life Technologies) for 15 minutes at 37°C, in order to further dissociate the cell sheets into a single cell suspension. Ham’s F-12 media was added to the tube, and the cells were again separated via centrifugation at the same settings. The supernatant was removed, and the cells were resuspended in 6 mL of Ham’s F-12 media containing 10% FBS before being seeded in Costar^®^ Transwell^®^ (12 mm, 0.4 μm pores) 12-well plates.

The media used to culture the mouse tracheal epithelial cells (MTECs) consisted of Ham’s F-12 media supplemented with 250 ng/ml amphotericin B solution (Hyclone), 20 ng/ml cholera toxin (LIST biological), 104 μg/ml bovine pituitary extract (Lonza), 5 μg/ml insulin (Sigma), 5 μg/ml apo-transferrin (Sigma), 0.1uM dexamethasone (Sigma), 5ng/ml mouse epidermal growth factor (Sigma), 0.01 μM retinol (Sigma), 20 U/ml nystatin (Sigma), 50 μg/ml gentamicin. During the growth and expansion phase of culture, this media was further supplemented with 10% FBS.

Prior to seeding of MTECs, transwell membranes were coated with 300ug/mL rat tail collagen in 0.02 N glacial acetic acid at room temperature for 1 hour. Excess liquid was then aspirated, and the transwell membranes were washed with sterile PBS. The transwells were then conditioned with Ham’s F-12 media on both the apical and basal sides for a minimum of 1 hour at 37°C. Prior to seeding of MTEC’s, this conditioning media was removed from both compartments, and 1mL of culture media containing 10% FBS was placed on the basolateral side of each well. Finally, 0.5 mL of the final cell suspension, mentioned previously, was placed on the apical side of the transwell. The plates were incubated at 37°C in a 5% CO_2_ atmosphere for 72 hours to allow for settling and adherence of MTEC’s to the coated membranes. After this period of time, the media from the apical and basal compartments was replaced every 48 hours with fresh, Ham’s F-12 media with 10% FBS. The cells were then monitored daily for confluence. When 100% confluence was reached (typically 7–10 days), media was removed from the apical side completely to establish an air-liquid interface (ALI), and the media on the basal side was changed to serum-free Ham’s F-12 culture media, replaced at 24-hour intervals. The cells were then maintained at ALI for a minimum of 14 days in order to reach maximum differentiation. Trans-epithelial electrical resistance (TEER) measurements were taken as a primary output to monitor cellular health and barrier integrity. MTEC culture media was added to the apical side of all wells for 30 minutes prior to baseline TEER measurements being recorded. This is a standard protocol for harvesting and culturing MTECs, which has been previously described by our laboratory [7].

### Statistics

All statistics were done using Graphpad Prism software. Since there were four genetically different groups of mice in these studies, two-way ANOVA with Bonferroni’s multiple comparison test was used to examine significant differences in the ozone exposed mice. One-way ANOVA was used to compare ozone exposed with filtered air controls. Student’s t-test were used when only comparing two groups independently. For flexivent analysis, values collected on separate experimental days were log-transformed prior to statistical analysis to account for daily variation of the methacholine challenges as previously described [14].

## Results

### Genetic variation in SP-A2 on airway resistance changes after exposure to ozone

**Fig 1:**
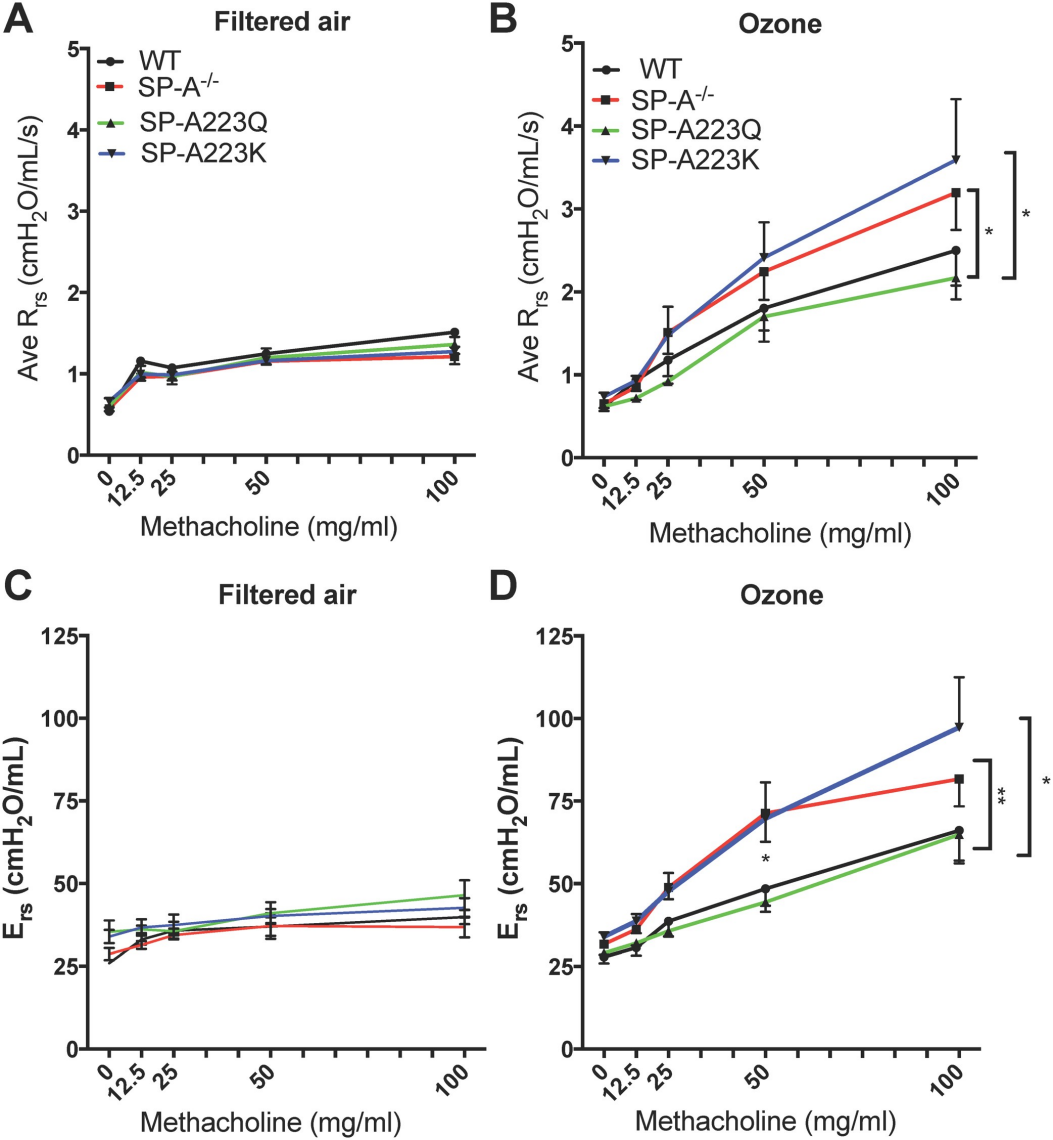
Genetic differences in SP-A2 on the physiological response to ozone at 24 hrs. Age-matched male mice were exposed to FA or 2 ppm Ozone (O_3_) for 3 hrs and airway reactivity (AHR) to methacholine was assessed 24 hrs post challenge. Data are presented as the average of the raw respiratory system resistance (Rrs) values for (**A**) FA and (**B**) O_3_ exposed mice and the average total elastance of the respiratory system (Ers) for (**C**) FA and (**D**) O_3_ exposed mice. FA controls n = 4 mice/genotype; O_3_ n = 6–8 mice/genotype. Graphs are representative of two independent experiments. FA controls vs O_3_ exposed by respective genotype, **WT (black lines)**: Rrs O_3_ exposed vs FA exposed (100 mg/ml MCH challenge,*p = 0.014); **SP**-**A**^**-/-**^ **(red lines)**: Rrs O_3_ exposed vs FA exposed (100 mg/ml MCH challenge, **p = 0.0015; 50 mg/ml MCH challenge, *p = 0.017); **SP**-**A223Q (green lines)**: Rrs O_3_ exposed vs FA exposed (100 mg/ml MCH challenge, *p = 0.03); **SP**-**A223K (blue lines)**: Rrs O_3_ exposed vs FA exposed (100 mg/ml MCH challenge, **p = 0.0083; 50 mg/ml MCH challenge, **p = 0.0047). O_3_ exposed vs O_3_ exposed of each genotype: **For** **Rrs**, WT vs SP-A223Q, 100 mg/ml dose p = 0.06; SP-A^-/-^ vs 223Q, 100 mg/ml dose *p = 0.03; SP-A223Q vs SP-A223K, 100 mg/ml dose *p = 0.03. **For** **Ers**, WT vs SP-A^-/-^, 50 mg/ml dose *p = 0.049; SP-A223Q vs SP-A^-/-^, at 50 mg/ml dose **p = 0.0094; SP-A223Q vs SP-A223K, 50 mg/ml dose *p = 0.035.

We first assessed the resistance of the entire respiratory system (Rrs). There was no significant difference in the sensitivity to methacholine challenge between the filtered air (FA) controls including WT, SP-A KO, SP-A223Q and SP-A223K mice (p = 0.57, r^2^ = 0.15, n = 4/genotype) by one-way ANOVA (Fig 1A and 1B). When compared to their respective FA controls, ozone (O_3_) exposure led to increased sensitivity to methacholine challenge in all genotypes of mice (Fig 1A and 1B). In WT O_3_ exposed mice, total airways resistance was significantly greater only at the highest 100 mg/ml MCH challenge point (*p = 0.014) as compared to FA controls. In contrast, mice completely lacking SP-A (SP-A^-/-^) that were O_3_ exposed had significantly increased total airways resistance at the 50 mg/ml (*p = 0.017) and 100 mg/ml (**p = 0.0015) MCH challenges as compared to respective FA controls.

Overall, the SP-A223Q humanized mice responded similar to WT mice with significantly increased total airways resistance only at the 100 mg/ml (*p = 0.03) dose of MCH as compared to FA controls. In contrast, SP-A223K humanized mice responded similar to SP-A^-/-^ mice with significantly greater total airways resistance at both the 50 mg/ml (**p = 0.0047) and 100 mg/ml MCH challenge (**p = 0.0083) as compared to FA controls.

When all four genotypes are assessed for total airways resistance at the highest 100 mg/ml dose of MCH across genotypes, the SP-A223Q O_3_ exposed mice had a significantly attenuated response as compared to both the SP-A^-/-^ O_3_ exposed mice (*p = 0.03) and the SP-A223K O_3_ exposed mice (*p = 0.03). This suggests that expression of SP-A2 223Q may be more protective from acute exposure to O_3_ as compared to expression of SP-A2 223K in regards to airway hyperresponsiveness.

In addition to total airways resistance, we also assessed the respiratory system elastance (Ers) across the four genotypes of mice. Ers is a measure of the overall stiffness of the entire respiratory system during tidal breathing. The trends were similar to what was observed for Rrs in that O_3_ exposed SP-A^-/-^ and SP-A223K mice had overall higher measures of Ers as compared to WT and SP-A223Q O_3_ exposed mice. When assessed at the 50 mg/ml dose of MCH, SP-A223Q mice had a significantly lower Ers measure as compared to SP-A^-/-^ (**p = 0.0094) and SP-A223K (*p = 0.035) mice. In addition, O_3_ exposed SP-A^-/-^ mice had significantly greater Ers as compared to WT O_3_ exposed mice at this dose (*p = 0.049). All O_3_ exposed mice had greater Ers as compared to their FA controls at the highest 100 mg/ml MCH dose (Fig 1C).

### Genetic variation in SP-A2 on airway inflammatory cell recruitment after exposure to ozone

**Fig 2:**
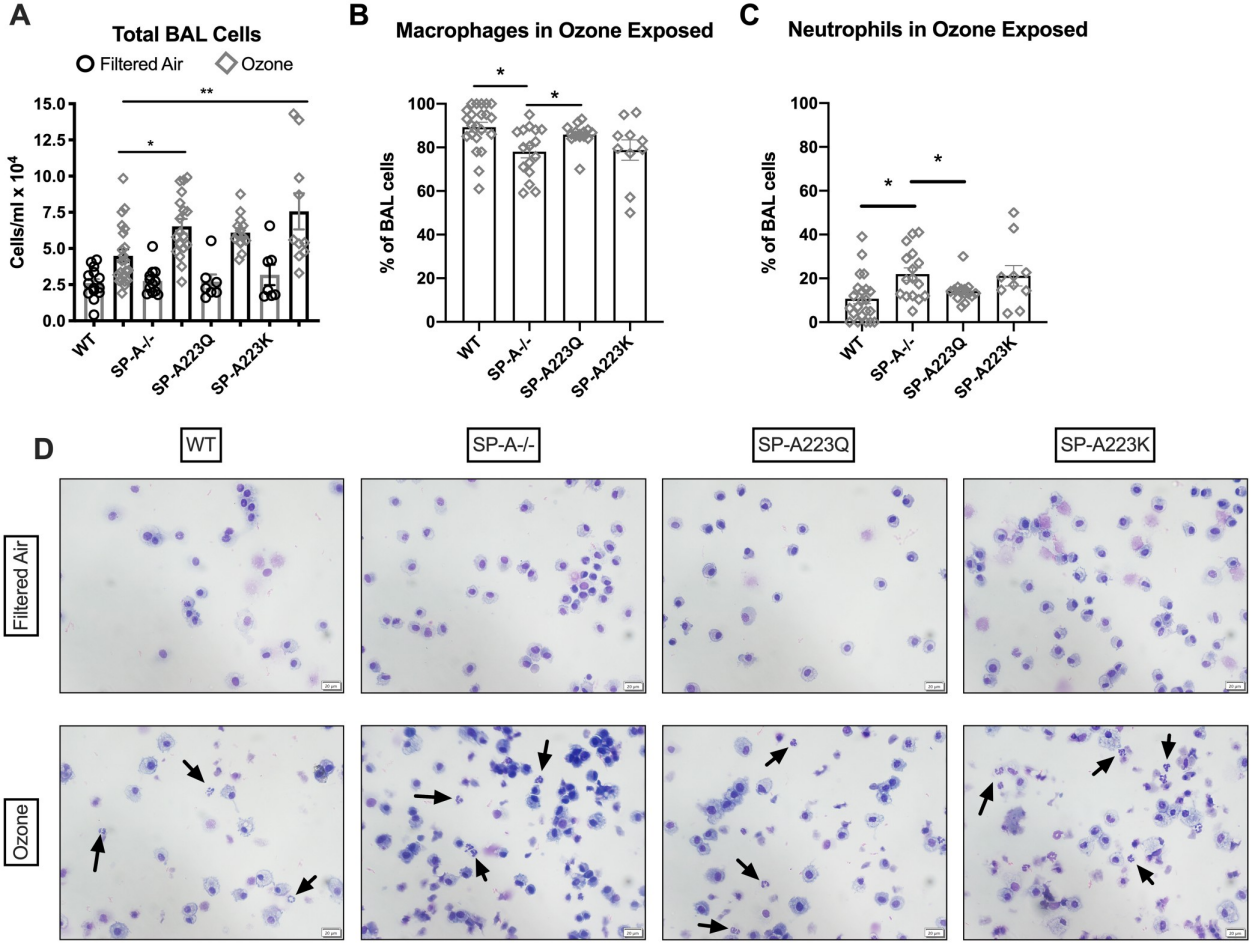
Genetic differences in SP-A2 on the cellular response to ozone at 23 hrs. After pulmonary function assessment, the lungs were gently lavaged and cellularity was determined. (**A**) Total cells were counted with Trypan blue exclusion for dead cells. The percentage of (**B**) macrophages and (**C**) neutrophils were determined 24 hrs post ozone challenge by differential cell counts. FA controls had ~98% macrophages (not shown). *p<0.05, **p<0.01. (**D**) Representative pictures of differentials take at 40X magnification, scale bar = 20 μm. Arrows indicate neutrophils.

After mice were examined for pulmonary function on the flexivent, the lungs were gently lavaged and total cells and differential cells were examined. There were no differences observed in the total cells recovered from BAL of the four genotypes of mice after FA exposure (Fig 2A). From the FA lavages, cells consisted of ~98% or higher macrophages in all four groups of mice. After O_3_ exposure, only SP-A^-/-^ mice and SP-A223K mice had significant increases in the total cells in the BALF as compared to WT mice (Fig 2A). When assessing the percentage of each cell type in the BALF, SP-A^-/-^ mice had a significantly higher percentage of neutrophils as compared to WT and SP-A223Q mice (Fig 2B).

**Fig 3:**
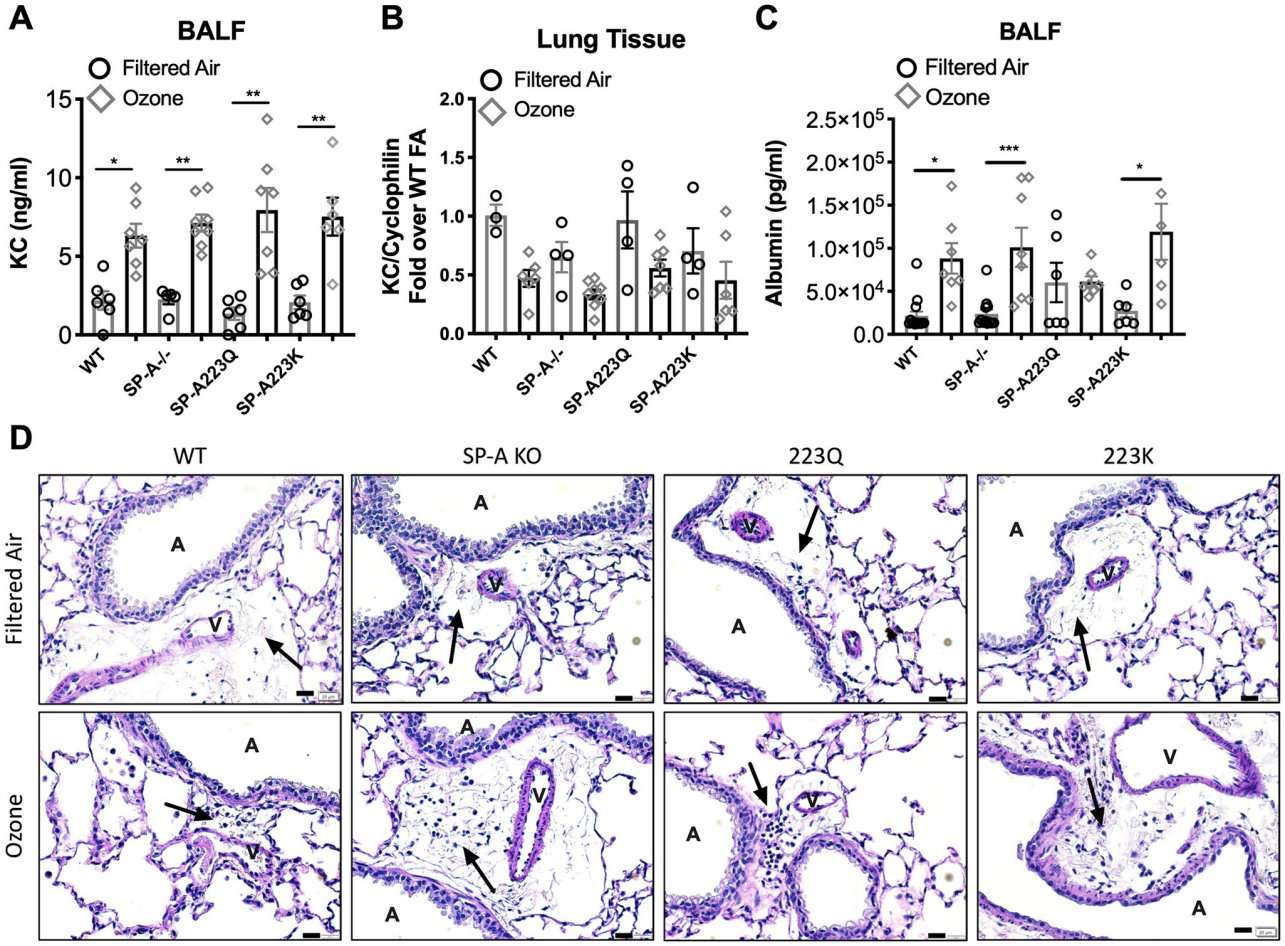
Genetic variation in SP-A2 on vascular permeability after exposure to ozone. **(A**) KC levels in BAL 24 hrs post ozone exposure. (**B**) KC gene expression in the lung tissue 24 hrs post ozone exposure. (**C**) Albumin protein levels in lavage fluid from O_3_ vs FA exposed mice. (**D**) Representative histology of lung sections depicting a vessel adjacent to an airway and surrounding lymphatic with cellular infiltration (arrows) of mice treated with FA (upper panel) or O_3_ (lower panel). Scale bar = 20 μm. V = vessel, A = airway. *p<0.05, **p<0.01, ***p<0.001.

In order to determine if neutrophil differences across the genotypes were due to differential regulation of cytokine recruitment, KC levels were assessed in BALF and in lung tissue. While KC levels in BALF were quite low, the levels were significantly elevated in O_3_ exposed mice as compared to their respective FA controls. However, there were no differences in KC levels in BAL between the 4 genotypes of mice (Fig 3A). Gene expression of KC in the lung tissue was not up-regulated at this time point (Fig 3B).

### Genetic variation in SP-A2 on vascular permeability and lung histology after exposure to ozone

In order to determine if genetic variation in SP-A2 had an impact on ozone-induced vascular permeability and edema, albumin levels were measured in the BALF from all groups of mice post FA or O_3_ exposure. While WT, SP-A^-/-^ and SP-A223K mice had significant increases in albumin in BALF post O_3_ exposure, O_3_ exposed SP-A223Q mice had BALF albumin levels that were not different from their FA controls (Fig 3C). While few leukocytes were observed in histological sections of FA treated mice, more cellular infiltration was evident in areas of lymphatics that were in close proximity to vessels and airways in all O_3_ exposed groups of mice (Fig 3D). Additionally, histology sections for all genotypes were examined for differences in the size of alveolar spaces, but there were no differences between the any of the genotypes or treatment groups. While models of chronic ozone exposure in mice have led to an increase in alveolar space diameter, models of acute ozone exposure, such as the one performed in this study, have not demonstrated any changes in this feature of lung tissue [15].

### Genetic variation in SP-A2 on epithelial cell integrity

**Fig 4:**
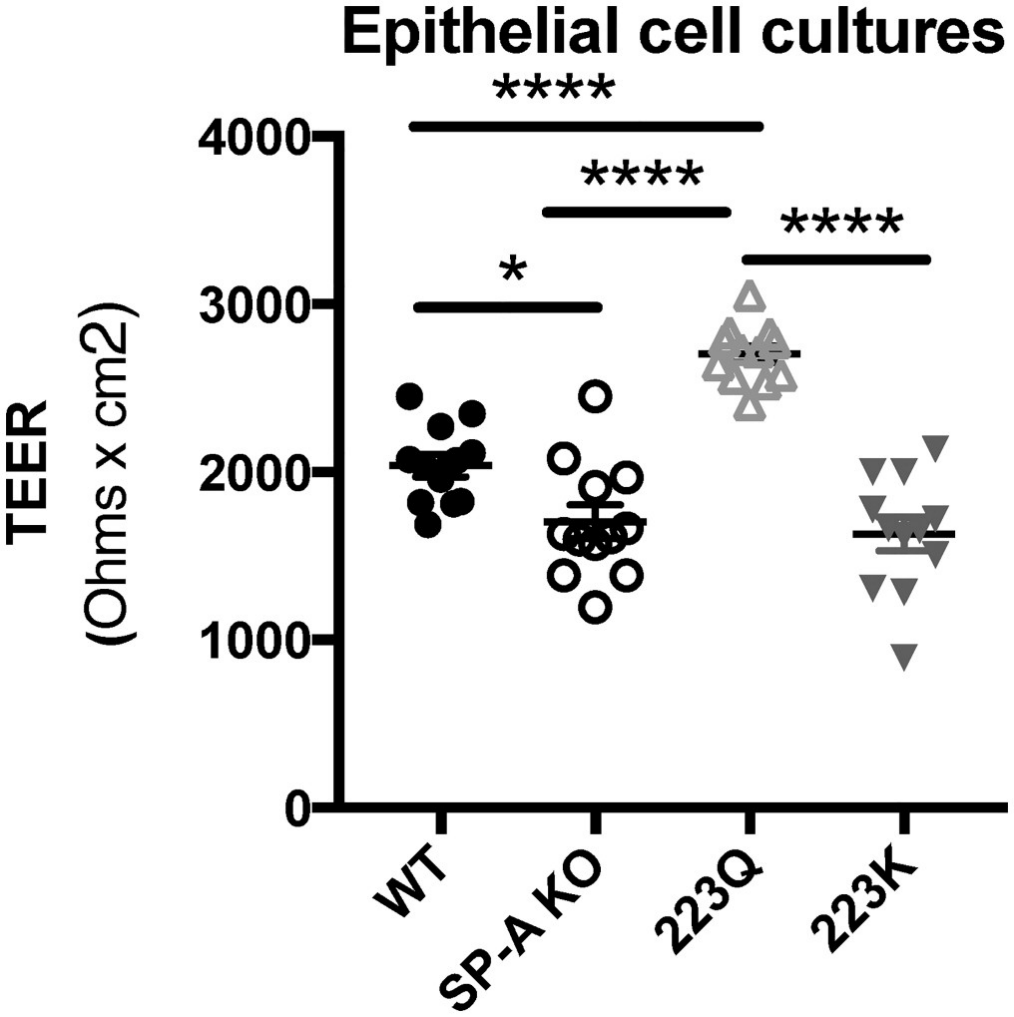
Genetic differences in SP-A2 may impact epithelial cell integrity. Trans-epithelial electrical resistance (TEER) levels of naive mouse tracheal epithelial cells (MTECS). n = 2 experimental repeats combined for total of 12 wells per genotype. Statistical analysis by One-way ANOVA: SP-A 223Q vs. WT, SP-AKO, & SP-A223K ***p < 0.0001. WT vs. SP-A KO *p < 0.05, WT vs SP-A 223K **p < 0.01.

Since SP-A223Q mice had the lowest change in albumin levels after O3 challenge, we sought to determine if epithelial cells grown at an air-liquid interface had similar or different measures of epithelial integrity with respect to the four genotypes of expressed SP-A. All cells were seeded and grown under identical conditions for the same amount of time. After spending two weeks at an air-liquid interface, TEER measurements were recorded from untreated MTEC cell cultures for all four genotypes. SP-A 223Q cells had significantly higher TEER measurements than all three other genotypes, for which all had a p-value < 0.0001. Wild Type cells also has significantly higher TEER measurements than SP-A KO (p = 0.0319) and SP-A 223K (p = 0.0060) cells. Interestingly, there was no difference between the TEER measurements of transwells containing SP-AKO or SP-A 223K cells (Fig 4).

## Discussion

SP-A is a key immune modulator in the lungs and has much relevance for its role in protecting from various lung diseases. While many publications highlight a role for SP-A in host defense against pathogens (reviewed in [16]), SP-A has been described as an important mediator in a variety of diseases including cystic fibrosis [17, 18], allergic rhinitis [19], sinusitis [20], lung cancer metastasis [21], pulmonary fibrosis [22], and more recently asthma ([3, 23, 24]) and COPD [25, 26]. Given the immense importance of SP-A in the lung, whether by participating directly or mediating indirectly, one can appreciate the need to have an adequate pool of functional SP-A for normal lung homeostasis. This study has shown that variation in the SP-A2 gene can impact the ability of SP-A to protect the lungs from the harmful effects of ozone exposure.

In this vein, our previous studies have shown that a subset of asthmatics, those obese individuals with BMI>30 have significantly reduced levels of SP-A as compared to non-obese asthmatics [23]. This represents a population with an inadequate pool of functional SP-A. In contrast, others have discovered that SP-A extracted from some asthmatic individuals is less functional than SP-A extracted from non-asthmatic individuals [24]. This would represent a population of individuals with an adequate amount of dysfunctional SP-A. When we consider reasons behind the dysfunction, we are led to the notion that not all of the genetic variation within SP-A that is expressed in the population would function identically. In addition, genetic differences that result in amino acid substitutions with regions of SP-A may alter the stability of the oligomeric structure and render it more susceptible to degrading factors found in the environment or to an inflammatory milieu.

According to Wang *et al*. [4], despite the amino acid substitution in SP-A2 at position Q223K, these 1A^0^ and 1A^3^ SP-A variants respectively, maintain a 99.6 percent protein identity. In the general population, the presence of at least one K allele is relatively low (~20–25%). However, among African Americans, the frequency is thought to be as high as ~35% [27]. While the prevalence of SP-A2 homozygosity for the K allele is less than 8% in the general population, homozygosity is over 12% in the African American subgroup [27]. Therefore this higher frequency of a less protective SP-A allele within the African American population may be a contributing factor to the increased incidence and severity of asthma in this subgroup and should be further explored [28]. Additionally, in a recent study Dy *et al* demonstrated that SP-A is involved in the resolution of allergic airway inflammation by inducing eosinophil apoptosis, and expression of the 223K variant in SP-A2 results in diminished SP-A functionality and would therefor likely impact asthma [29].

Others have also described findings in which specific SP-A haplotypes are associated with disease. In a study of infants, the 6A allele haplotype of *SFTPA1* was associated with an increased risk of persistent cough and wheeze [30]. Additional polymorphisms within *SFTPA1* and *SFTPA2*, have been linked to respiratory distress syndrome in infants [31–33], severe RSV bronchiolitis [34], and otitis media [35, 36]. While little is known about how the genetic heterogeneity within SP-A1 and SP-A2 affects the levels of protein expression in human lung BALF, we know that human SP-A2 expression is similar between the two groups of humanized mice used in our study at both the gene expression and protein level [7]. So this rules out differences in expression between SP-A2 223Q and SP-A2 223K as the cause of the differential response to ozone exposure.

Haque *et al* showed similar responses of SP-A^-/-^ mice as compared to WT mice in an ozone exposure model with significantly increased total cells and protein in BAL at 24 hours post exposure [37]. Their work showed a dramatic spike in neutrophils in WT mice within the first 4 hours post ozone exposure. Studies by Laskin *et al*. also demonstrated acute neutrophilic inflammation in the lungs of WT mice 3 hours after exposure to even lower concentrations of ozone (0.8ppm) [38, 39]. While the neutrophilia observed in this study at 24 hours could indicate a more severe inflammatory response as a result of higher ozone levels, it is also possible that SP-A is involved in the resolution of the inflammation and damage caused by ozone. Even though we did not measure additional timepoints or ozone concentrations, one can appreciate that this very early response may be due to the inflammatory state of an ozone-oxidized SP-A that would potentially have subsided by 24 hours with the presence of newly secreted SP-A in WT mice. Since we detected differences in neutrophils based on SP-A genotypes, we chose to further examine the predominant cytokine that would most likely be recruiting them, KC. While we found all groups exposed to ozone had significantly increased KC levels in BALF, there were no differences between the genotypes. In addition, the mice used in the previous study were younger (5–6 weeks) during the ozone exposure as compared to our 8-10-week-old mice that we used, which could contribute to the slight differences we detected in the neutrophil response.

Another mechanism that could contribute to increased neutrophil infiltration into the lumen of the lung is through disruption of the epithelial layer and increased vascular permeability. This phenomenon has been previously described to be enhanced with ozone exposure [40]. Inhaled ozone impairs the function of tight junctions, adherens junctions, and many other proteins that are important players in barrier function of the epithelial layer [41]. With these proteins impaired, the integrity of the epithelial layer would be severely compromised, and albumin from the blood leaks from the vasculature into the pulmonary airspace, allowing for increased levels to be detected in BAL. So, vascular permeability is often determined by detection of the serum protein albumin in the BALF. We did detect significant differences in albumin levels in the BALF of SP-A-/- and SP-A223K mice after ozone exposure as compared to FA controls. However, ozone exposed SP-A223Q mice did not have any detectable differences from their FA controls, indicating a level of protection from ozone-induced vascular permeability not seen in the other genotypes.

We thought it surprising that we did not detect any changes in the albumin levels in BAL of O_3_ exposed SP-A223Q mice, while all other genotypes had increases post exposure. Normally, albumin is detectable in the serum, with very low levels in the BAL of healthy mice. A widely accepted method of measuring the level of impairment of tight junction dynamics, and consequent epithelial cell barrier function, in culture is transepithelial electrical resistance (TEER) [42]. Interestingly, we discovered that tracheal epithelial cells from the SP-A223Q mice had the highest level of epithelial integrity as measured by TEER measurements in an unchallenged state. While we were unable to determine the effect of ozone exposure on MTEC cultures due to insufficient resources, a significantly higher epithelial barrier integrity at baseline could offer better initial protection, which could be a contributing factor to the unaltered albumin levels post exposure. The superior epithelial integrity displayed by the SP-A223Q cells, at baseline, suggests an interaction between functional SP-A proteins and the barrier function of the epithelial layer of the airways. This, in turn, could be a contributing factor to the body’s ability to respond to inhaled insults. A better understanding of how SP-A223Q contributes mechanistically to the formation and maintenance of gap junctions in the epithelium warrants more investigation.

A limitation of our study is the use of exclusively male mice for our physiological assessments. Although we recognize the importance to study the effects of ozone exposure in relation to SP-A genetic variation in females as well as males, recent reports demonstrate that females have a much less consistent physiologic response to ozone and highly variable measures of AHR [8]. We therefore chose to examine the effect of SP-A2 variation in male mice only for these initial studies. Additionally, our mice were only exposed to a one-time acute challenge with O_3_, whereas environmentally, humans are exposed to high O_3_ in a more chronic manner. While our studies only offer a snapshot into how genetic variation in SP-A2 alters the physiologic and immunologic response to acute O_3_, future studies are needed to better understand the contributing mechanisms to the phenotypes that we have observed.

As more individuals are affected by poor air quality and increased O_3_ exposure around the world, these studies are relevant in that we provide new evidence suggesting that individuals harboring specific genetic variation within the minor allele of SP-A2, SP-A223K, may be more susceptible to O_3_-induced negative lung phenotypes than others expressing the major allele SP-A223Q.

## Supporting information

S1 Table (DOCX)

S1 Data (XLSX)
