# Supplementary material for: Genetic variation in surfactant protein-A2 alters responses to ozone
Source: PLoS One. 2021 Feb 22;16(2):e0247504. doi: 10.1371/journal.pone.0247504 (PMC7899376; doi:10.1371/journal.pone.0247504)
Supplement: S1 Table — (DOCX) [file pone.0247504.s001.docx]

| **Experiment N Values for mice included in pulmonary function tests;**  **Values from one representative experiment are shown.** | | | | |
| --- | --- | --- | --- | --- |
| **Treatment** | **Wild Type** | **SP-A KO** | **SP-A 223Q** | **SP-A 223K** |
| **Filtered Air** | 4 | 4 | 4 | 4 |
| **Ozone** | 7 | 8 | 7 | 6 |
| **Experiment N Values for mice included in inflammatory readouts;**  **All values from two independent experiments were combined.** | | | | |
| **Treatment** | **Wild Type** | **SP-A KO** | **SP-A 223Q** | **SP-A 223K** |
| **Filtered Air** | 14 | 12 | 7 | 7 |
| **Ozone** | 22 | 18 | 14 | 10 |
